# Germline mutations in homologous recombination repair genes among Chinese pancreatic ductal adenocarcinoma patients detected using next‐generation sequencing

**DOI:** 10.1002/mgg3.2170

**Published:** 2023-03-28

**Authors:** Huiqin Jiang, Fei Huang, Xinning Chen, Li Zhang, Minna Shen, Baishen Pan, Beili Wang, Wei Guo

**Affiliations:** ^1^ Department of Laboratory Medicine Zhongshan Hospital, Fudan University Shanghai China; ^2^ Branch of National Clinical Research Center for Laboratory Medicine Shanghai China; ^3^ Department of Laboratory Medicine, Xiamen Branch Zhongshan Hospital, Fudan University Xiamen China

**Keywords:** germline mutations, homologous recombination, next‐generation sequencing, pancreatic ductal adenocarcinoma

## Abstract

**Background:**

Genetic testing for pancreatic ductal adenocarcinoma (PDAC) patients is in constant development. However, the status of homologous recombination repair (HRR) genes in unselected Chinese PDAC has not been fully explored. This study aims to characterize the profile of germline mutations in HRR genes in Chinese PDAC patients.

**Methods:**

A cohort of 256 PDAC patients were enrolled at Zhongshan Hospital Fudan University between 2019 and 2021. Germline DNA was analyzed by next‐generation sequencing using a multigene panel of the 21 HRR genes.

**Results:**

The germline pathogenic (P)/likely pathogenic (LP) variant rates were 7.0% (18/256) in unselected patients with pancreatic cancer. Among them, 1.6% (4/256) were identified as harboring *BRCA2* variants, and 5.5% (14/256) patients carried non‐BRCA variants. Variants were detected in eight non‐BRCA genes, including *ATM* (4, 1.6%), *PALB2* (4, 1.6%), *ATR* (1, 0.4%), *BRIP1* (1, 0.4%), *CHEK2* (1, 0.4%), *MRE11* (1, 0.4%), *PTEN* (1, 0.4%), and *STK11* (1, 0.4%). *ATM*, *BRCA2*, and *PALB2* were the most prevalent variant genes. If only *BRCA1/2* was tested, 5.5% of P/LP variants would have been lost. Further, we found that the landscape of P/LP HRR variants in various population cohorts was quite different. However, no significant difference was found in clinical characteristics between germline HRR P/LP carriers and non‐carriers. In our study, one case carrying a germline *PALB2* variant showed a long‐time response to platinum‐based chemotherapy and PARP inhibitor.

**Conclusion:**

This study comprehensively depicts the prevalence and characteristics of germline HRR mutations in unselected Chinese PDAC patients. Our findings show the clinical utility of a multigene panel may increase the detection of P/LP HRR carriers.

## Graphical abstract

ATM, BRCA2, and PALB2 were the most prevalent mutation genes in Chinese patients with pancreatic ductal adenocarcinoma (PDAC). An additional 5.8% of patients were defined to harbor pathogenic /likely pathogenic variations in other genes beyond BRCA1/2. A multi‐gene panel of HRR genes has a potential value in improving clinical management o PDAC patients.

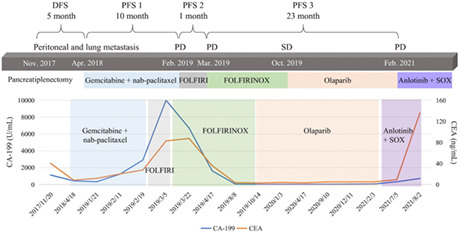

## 1 INTRODUCTION

Pancreatic ductal adenocarcinoma (PDAC) represents the most common form of pancreatic cancer. It is the seventh leading cause of global cancer death, with an estimated 9% 5‐year survival rate (Grossberg et al., 2020). The limited therapeutic option is one of the key factors that contribute to poor outcomes for PDAC patients. Recent studies are focused on identifying new therapeutic targets and investigating the genomic landscape of PDAC (Lai et al., 2021).

Genetic testing for PDAC patients is in constant development, and specific genetic alterations indicate personalized managing strategies. Germline pathogenic (P)/likely pathogenic (LP) *BRCA1/2* variants are associated with significantly increased risk of PDAC, which are found in approximately 5%–10% of cases of familial PDAC and approximately 3% of cases of apparently sporadic PDAC (Blair et al., 2018). Poly(ADP‐ribose) Polymerase (PARP) inhibitors have shown remarkable efficacy in the clinical management of several *BRCA*‐mutated tumors. The phase III trial showed that maintenance treatment with olaparib after chemotherapy lead to a significantly longer progression‐free survival compared with placebo in PDAC with germline *BRCA1* and *BRCA2* (*gBRCA1/2*) variants (7.4 months vs. 3.8 months, *p* = 0.004, HR = 0.53) (Golan et al., 2019). According to this study, olaparib was approved by FDA as a maintenance treatment for patients with P/LP variants in *gBRCA1/2*, and platinum‐sensitive, metastatic pancreatic adenocarcinoma. However, *gBRCA1/2* variants are found in approximately 5%–10% of cases of familial PDAC and approximately 3% of cases of apparently sporadic PDAC (Blair et al., 2018).

Emerging evidence suggests that patients with P/LP variants in several additional homologous recombination repair (HRR) related genes (e.g., ATM, PALB2, ATR, RAD 51, and CHEK2) might benefit from PARP inhibitors (Reiss et al., 2021; Urbina‐Jara et al., 2019). Recently, a Phase II Study (NCT03140670) investigating rucaparib, another PARP inhibitor, showed promising efficacy in patients with *BRCA1/2* as well as *PALB2* variants (Reiss et al., 2021). Hence, panel genetic testing of germline P/LP HRR variants is meaningful to identify PDAC patients who will derive clinical benefits from PARP inhibition. On the other hand, germline P/LP variants of DNA repair genes have been associated with an increased risk for PDAC and other tumors (Hu et al., 2018; Yang et al., 2020). Therefore, the National Comprehensive Cancer Network (NCCN) currently recommends genetic counseling and genetic testing to all patients with PDAC as well as to first‐degree relatives of patients with PDAC.

However, the prevalence of germline HRR variants in the Chinese population is rarely reported. Herein, we applied next‐generation sequencing (NGS) technologies to test DNA from 256 whole blood samples. Germline deleterious variants, covering 22 core HRR genes, were identified in Chinese patients with PDAC, and the relationship between genetic alterations and clinical parameters was analyzed.

## 2 MATERIALS AND METHODS

### 2.1 Patients and samples

A cohort of 256 PDAC patients who were admitted to the Zhongshan Hospital Fudan University between June 2019 and June 2021 was included in the study. Clinical information was retrieved from medical records. The study complied with the ethical standards of the Declaration of Helsinki and was reviewed and approved by the institutional ethics committee (Zhongshan Hospital Fudan University; B2021‐056). Written informed consent was obtained from all participants.

### 2.2 Clinical characteristics collection

Demographic and clinical information, including age, gender, tumor location, tumor stage, and *KRAS* status of tumor tissue were retrieved from the hospital information system. The definition of family history is these first‐degree relatives with a history of any solid malignancy.

### 2.3 NGS sequencing

A 10‐mL blood sample was collected from each patient in an EDTA blood collection tube. Germline DNA (gDNA) was extracted using Blood DNA kit (Amoy Diagnostics) and quantified using Qubit (Life Technologies). Germline DNA was analyzed using a handle HRR NGS panel (Amoy Diagnostics) according to manufacturer's instructions, which covered single‐nucleotide variants (SNV) and insertions/deletions (Indel) variants in protein‐coding regions and intron/exon boundaries of 21 genes including *ATM* (NM_000051.3), *ATR* (NM_001184.3), *BARD1* (NM_000465.3), *BRCA1* (NM_007294.3), *BRCA2* (NM_000059.3), *BRIP1* (NM_032043.2), *CDH1* (NM_004360.3), *CDK12* (NM_016507.3), *CHEK1* (NM_001274.5), *CHEK2* (NM_007194.3), *FANCL* (NM_018062.3), *MRE11* (NM_005591.3), *NBN* (NM_002485.4), *PALB2* (NM_024675.3), *PTEN* (NM_000314.4), *RAD51B* (NM_133509.4), *RAD51C* (NM_058216.3), *RAD51D* (NM_002878.4), *RAD54L* (NM_001142548.1), *STK11* (NM_000455.4), and *TP53* (NM_000546.5). This panel allowed the detection of copy number variations (CNV) of *BRCA1/2*. Briefly, DNA libraries were constructed by molecular inversion probe‐based capture, and then sequenced to a depth of ×100 on the MiSeq platform (Illumina, San Diego, USA).

### 2.4 Variant calling and bioinformatics analysis

Sequence data were aligned to the UCSC hg19 human reference genome sequence. The interpretation of all identified variants was based on current evidence from the scientific literature and public databases, including ClinVar, Ensembl, Varsome, LOVD, dbSNP, Genome Aggregation Database (gnomAD), and Exome Aggregation Consortium (ExAC). The clinical classification of the variants was carried out according to the American College of Medical Genetics and Genomics (ACMG) recommendations with the 5‐tier system: benign (B), likely benign (LB), variant of uncertain significance (VUS), LP, and P (Richards et al., 2015).

### 2.5 Statistical analysis

The distribution of each categorical variable was summarized in terms of its frequencies and percentages. The comparisons of categorical data were done using Fisher's exact test. A *p*‐value <0.05 (2‐sided) was considered statistically significant. Statistical analysis was performed using Statistical Product and Service Solutions (SPSS) Statistics 20.0 (IBM Corporation).

## 3 RESULTS

### 3.1 Patient characteristics

From July 2019 to July 2021, a total of 256 Chinese PADC patients were included in this study. The median age at diagnosis was 64 years old (range 32–82), and 161 (62.9%) patients were male. Ninety‐one patients (35.5%) had signs of metastasis at the time of diagnosis, and 20 patients (7.8%) had a history of multiple cancer. Family history of cancer within third‐degree relatives was confirmed in nine patients (3.5%). Patient detailed clinicopathological characteristics are summarized in Table 1.

**TABLE 1:** Baseline characteristics of overall patients.

Variable	Overall *N* = 256	Pts without P/LPVs *N* = 238 (93.0%)	Pts with P/LPVs *N* = 18 (7.0%)	*p* value
Early onset (<50 years)				1.000
Yes	25	23 (92.0%)	2 (8.0%)	
No	231	215 (93.1%)	16 (6.9%)	
Gender				0.240
Male	161	152 (94.4%)	9 (5.6%)	
Female	95	86 (90.5%)	9 (9.5%)	
Location of primary tumor				0.951
Tail	86	80 (93.0%)	6 (7.0%)	
Others	167	155 (92.8%)	12 (7.2%)	
NA	3	3 (100%)	0 (0.0%)	
Metastasis				0.066
M0	165	157 (95.2%)	8 (4.8%)	
M1	91	81 (89.0%)	10 (11.0%)	
KRAS				1.000
MT	190	177 (93.2%)	13 (6.8%)	
WT	35	33 (94.3%)	2 (5.7%)	
NA	31	28 (90.3%)	3 (9.7%)	
Family history of cancer				0.250
Yes	9	7 (77.8%)	2 (22.2%)	
No	247	231 (93.5%)	16 (6.5%)	
Occurrence of multiple tumors				0.319
Yes	20	17 (85.0%)	3 (15.0%)	
No	236	221 (93.6%)	15 (6.4%)	

Abbreviations: MT, mutant‐type; P/LPV, pathogenic/likely pathogenic variant; WT, wild‐type.

### 3.2 Landscape of germline HRR gene alterations

**FIGURE 1:**
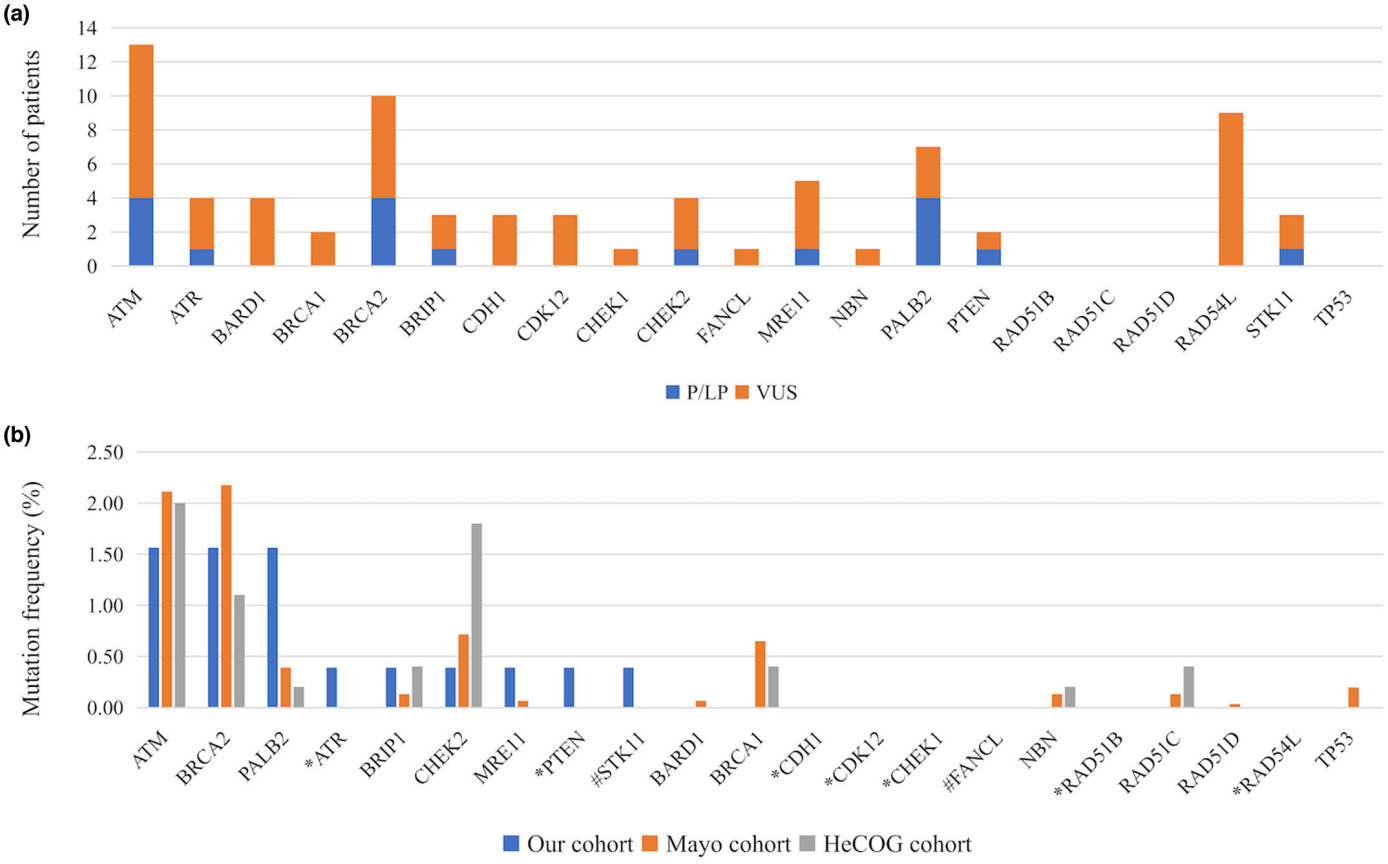
HRR gene mutation spectrum in our cohort. (a) Prevalence of HRR P/LP and VUS in 154 patients of the study. (b) Comparison of P/LP mutation frequency in PDAC patients between our cohort and other clinic cohort. *These genes were not included in the panel used by both mayo cohort and HeCOG cohort. ^#^These genes were not included in the panel used by mayo clinic cohort. LPV, likely pathogenic variant; P, pathogenic; VUS, variant of uncertain significance. *ATM* (NM_000051.3), *ATR* (NM_001184.3), *BARD1* (NM_000465.3), *BRCA1* (NM_007294.3), *BRCA2* (NM_000059.3), *BRIP1* (NM_032043.2), *CDH1* (NM_004360.3), *CDK12* (NM_016507.3), *CHEK1* (NM_001274.5), *CHEK2* (NM_007194.3), *FANCL* (NM_018062.3), *MRE11* (NM_005591.3), *NBN* (NM_002485.4), *PALB2* (NM_024675.3), *PTEN* (NM_000314.4), *RAD51B* (NM_133509.4), *RAD51C* (NM_058216.3), *RAD51D* (NM_002878.4), *RAD54L* (NM_001142548.1), *STK11* (NM_000455.4), *TP53* (NM_000546.5).

**FIGURE 2:**
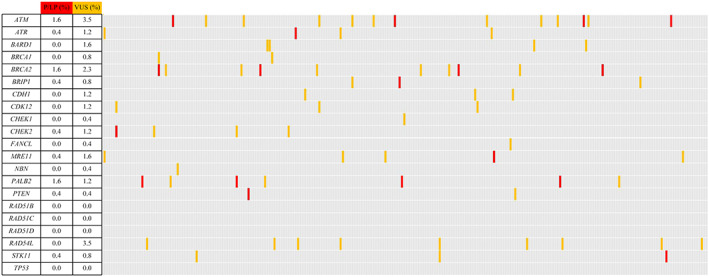
Landscape of germline HRR gene alterations of the 154 Chinese PDAC patients in our study. Each column represents one patient. LPV, likely pathogenic variant; P, pathogenic; VUS, variant of uncertain significance. *ATM* (NM_000051.3), *ATR* (NM_001184.3), *BARD1* (NM_000465.3), *BRCA1* (NM_007294.3), *BRCA2* (NM_000059.3), *BRIP1* (NM_032043.2), *CDH1* (NM_004360.3), *CDK12* (NM_016507.3), *CHEK1* (NM_001274.5), *CHEK2* (NM_007194.3), *FANCL* (NM_018062.3), *MRE11* (NM_005591.3), *NBN* (NM_002485.4), *PALB2* (NM_024675.3), *PTEN* (NM_000314.4), *RAD51B* (NM_133509.4), *RAD51C* (NM_058216.3), *RAD51D* (NM_002878.4), *RAD54L* (NM_001142548.1), *STK11* (NM_000455.4), *TP53* (NM_000546.5).

**FIGURE 3:**
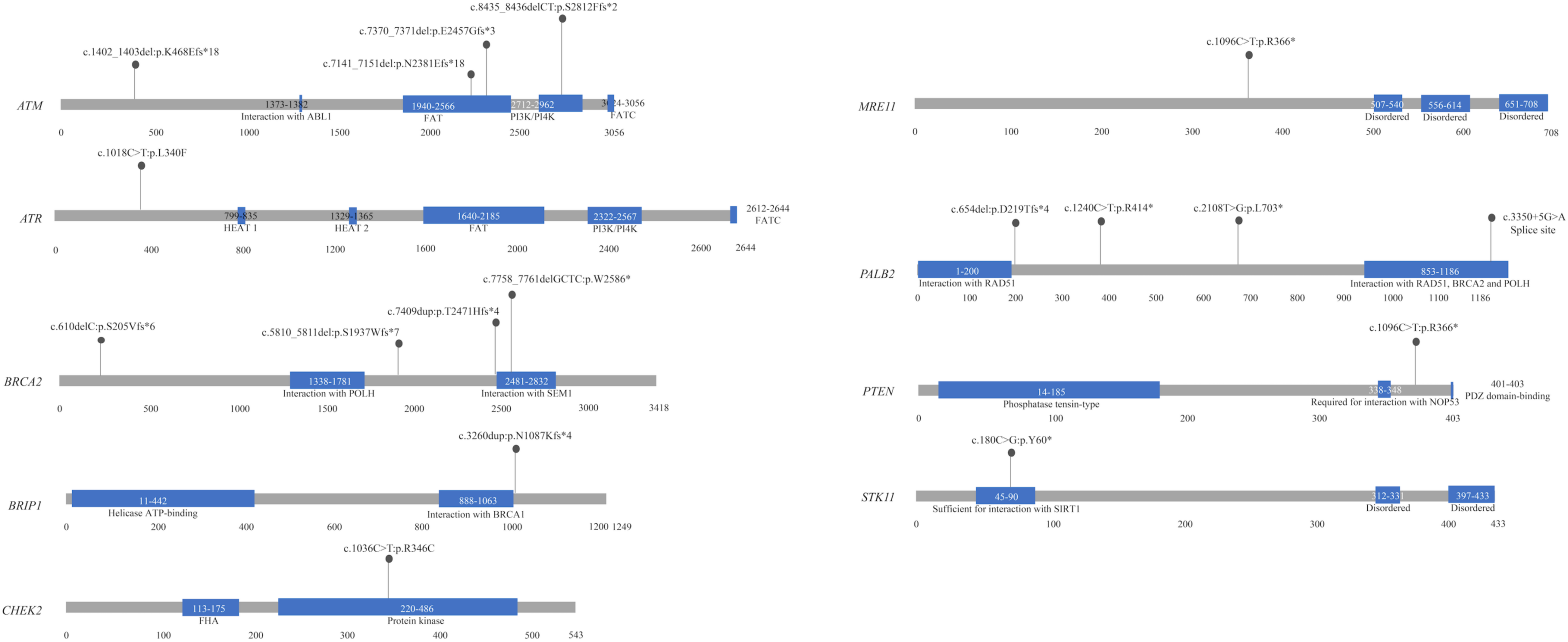
Locations of pathogenic germline mutations in HRR genes. Each mutation identified is shown by a lollipop plot. *ATM* (NM_000051.3), *ATR* (NM_001184.3), *BRCA2* (NM_000059.3), *BRIP1* (NM_032043.2), *CHEK2* (NM_007194.3), *MRE11* (NM_005591.3), *PALB2* (NM_024675.3), *PTEN* (NM_000314.4), *STK11* (NM_000455.4).

The overall landscape of unique alterations in each patient is summarized in Figure 1. HRR P/LP variants were identified in 7.0% (18/256) of patients in our study, whereas 53 patients were carriers of VUS (22.3%). The majority of P/LP variants occurred in *ATM*, *BRCA2*, and *PALB2* (Figure 2). Of the 18 P/LP variants, nine were frameshifts, six were nonsense SNVs, one were splice sites, and two were missense SNVs (Table 2, Figure 3). Most of these variants were absent or rare in East Asian from gnomAD database.

**TABLE 2:** Mutation details of P/LP gene variants.

ID	Gene	Variants	Function	Classification	Allele frequency in East Asian
8	*CHEK2*	NM_007194.3:exon10:c.1036C>T:p.R346C	Missense	LP	5.44E‐5
908270008	*PALB2*	NM_024675.3:exon5:c.2108T>G:p.L703*	Nonsense	P	5.44E‐5
909270023	*BRCA2*	NM_000059.3:exon16:c.7758_7761delGCTC:p.W2586*	Nonsense	LP	NR
910270021	*ATM*	NM_000051.3:exon58:c.8435_8436delCT:p.S2812Ffs*2	Frameshift	LP	1.64E‐4
2003270031	*PALB2*	NM_024675.3:exon4:c.1240C>T:p.R414*	Nonsense	P	0
2003270048	*PTEN*	NM_000314.4:exon7:c.664G>A:p.V222M	Missense	LP	NR
2003270057	*BRCA2*	NM_000059.3:exon7:c.610delC:p.S205Vfs*6	Frameshift	P	NR
2007270009	*ATR*	NM_001184.3:exon18:c.3547C>T:p.R1183*	Nonsense	LP	NR
2106270037	*ATM*	NM_000051.3:exon49:c.7141_7151del:p.N2381Efs*18	Frameshift	P	NR
2106270052	*BRIP1*	NM_032043.2:exon20:c.3260dupp.N1087Kfs*4	Frameshift	LP	0
2106270058	*PALB2*	NM_024675.3:intron12:c.3350+5G>A:p.?	Splice site	LP	0
2107270070	*BRCA2*	NM_000059.3:exon14:c.7409dup:p.T2471Hfs*4	Frameshift	P	NR
2108270010	*MRE11*	NM_005591.3:exon10:c.1096C>T:p.R366*	Nonsense	LP	NR
2109270046	*PALB2*	NM_024675.3:exon4:c.654del:p.D219Tfs*4	Frameshift	P	0
2110270001	*ATM*	NM_000051.3:exon50:c.7370_7371del:p.E2457Gfs*3	Frameshift	LP	NR
2110270014	*BRCA 2*	NM_000059.3:exon11:c.5810_5811del:p.S1937Wfs*7	Frameshift	LP	NR
2111270031	*STK11*	NM_000455.5:exon1:c.180C>G:p.Y60*	Nonsense	P	NR
2111270036	*ATM*	NM_000051.3:exon10:c.1402_1403del:p.K468Efs*18	Frameshift	P	3.01E‐4

Abbreviations: LPV, likely pathogenic variant; NR, not reported in gnomAD database; P, pathogenic. Data of allele frequency in population were obtained from gnomad database.

To determine the landscape of P/LP HRR variants in different populations, we compared variant frequencies among our cohort and other two cohorts (Figure 1) (Fountzilas et al., 2021; Hu et al., 2018). Of the 3078 patients included in the Mayo Clinic cohort, 209 (6.8%) were found to carry a variant in one of the 12 HRR genes (*ATM*, *BARD1*, *BRCA1*, *BRCA2*, *BRIP1*, *CHEK2*, *MRE11*, *NBN*, *PALB2*, *RAD51C*, *RAD51D*, and *TP53*). The most common alterations in the Mayo Clinic cohort were *BRCA2* (2.2%), *ATM* (2.1%), *BRCA1* (0.6%), and *PALB2* (0.4%). In Hellenic Cooperative Oncology Group (HeCOG) cohort, 6.6% (36/549) PDAC patients were found to carry a variant in one of the eight HRR genes (*ATM*, *BRCA1*, *BRCA2*, *BRIP1*, *CHEK2*, *NBN*, *PALB2*, and *RAD51C*), and the most common alterations occurred in *ATM* (2.0%), *CHEK2* (1.8%), *BRCA2* (1.1%), *BRIP1* (0.4%), *RAD51C* (0.4%), and *BRCA1* (0.4%). Similar germline HRR variant frequencies between our cohort and other two cohorts (7.4% vs. 6.8% vs. 6.6%) were observed. However, the variant frequencies in HRR genes involved in three cohort studies were quite different. Nine genes (*ATR*, *CDH1*, *CDK12*, *CHEK1*, *FANCL*, *PTEN*, *RAD51B*, *RAD54L*, and *STK11*) were not included in the panel used by either mayo clinic cohort or HeCOG cohort, and these genes contributed 1.6% of the variant rate in our cohort. Variants in six genes (*BRCA1*, *BARD1*, *NBN*, *RAD51C*, *RAD51D*, and *TP53*) were not observed in our study, and also at low frequency level in other two cohorts.

### 3.3 Correlations between clinical characteristics and P/LP HRR variant

HRR variants were observed more frequently in patients with a family history of cancer (including esophageal cancer, breast cancer, colorectal cancer, gastric cancer, lung cancer, pancreatic cancer, and liver cancer), whereas there was no significant difference (22.2% vs. 6.5%, *p* = 0.250). Of the 18 patients harboring P/LP HRR variants, only two had a family history of cancer. We found no statistically significant association between HRR mutation status and age, gender, *KRAS* status, tumor location, tumor status, or multiple cancer history (Table 1).

### 3.4 Germline *PALB2* variant is a promising biomarker for platinum‐based chemotherapy and PARP inhibitor

**FIGURE 4:**
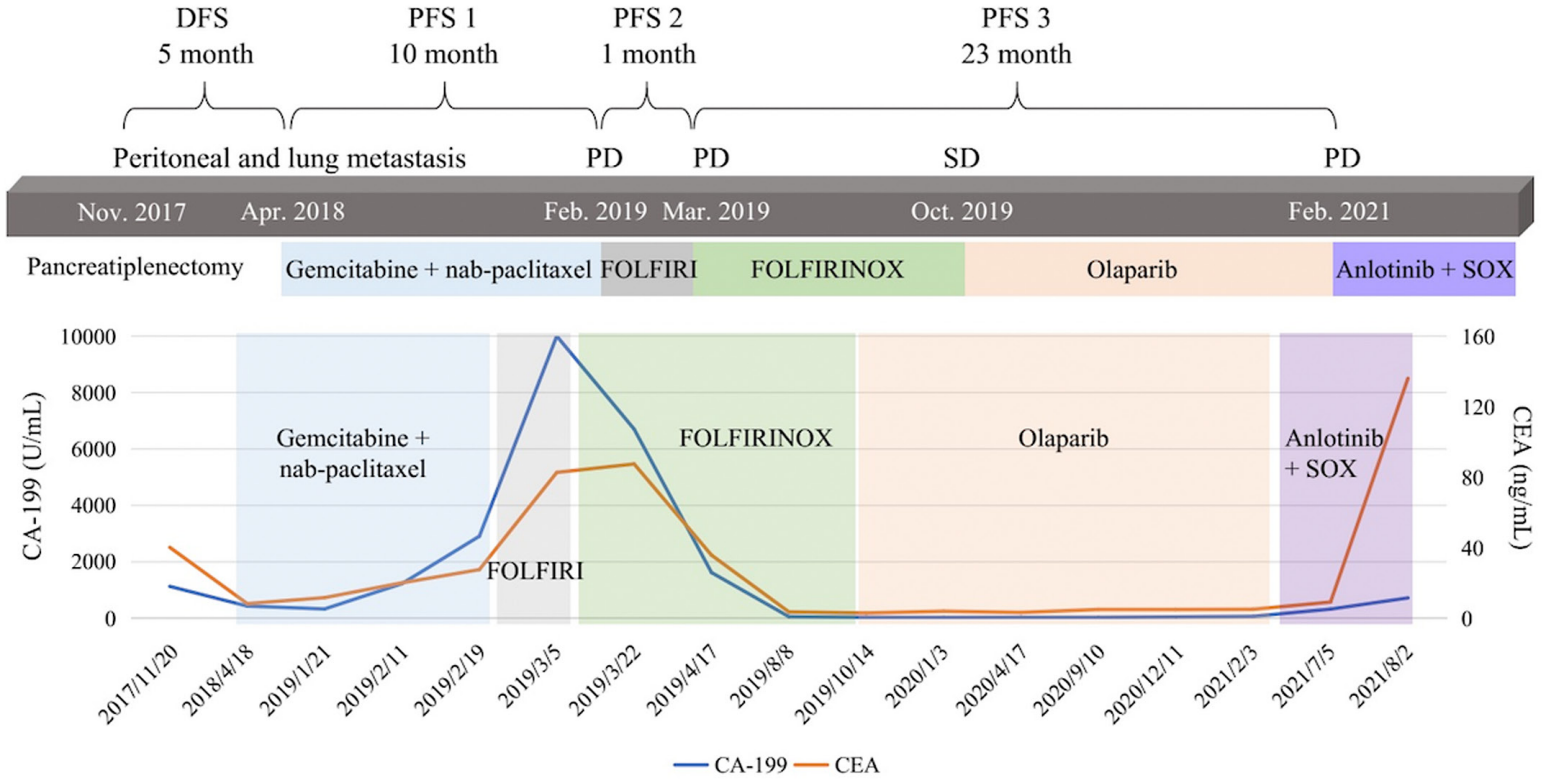
A 50‐year‐old male was diagnosed with pancreatic adenocarcinoma in July 2017. The patient underwent pancreatiplenectomy in November 2017. Five months postoperatively, multiple metastases appeared involving the lung and peritoneum. He received chemotherapy with gemcitabine and nab‐paclitaxel as first‐line therapy since Apr 2018. PET‐CT revealed PD on Feb 2019. Since then, he received 2 cycles of FOLFIRI. However, the patients underwent rapid PD. Then he received FOLFIRINOX as third‐line therapy, and follow‐up images revealed PR. Mutational profiling analysis using targeted next‐generation sequencing revealed that the patient carried a germline PALB2 mutation. Olaparib was thereby administered as maintenance therapy. The patient showed a significant response to Olaparib and a PFS of 23 months was observed. DFS, disease‐free survival; PD, progression disease; PFS, progression‐free survival; PR, partial response; SD, stable disease.

In this study, we observed that one patient with a germline *PALB2* variant exhibited a long‐time response to platinum‐based chemotherapy and PARP inhibitor (Figure 4). The patient underwent pancreatiplenectomy in November 2017. Five months postoperatively, multiple metastases appeared involving the lung and peritoneum. He received a standard gemcitabine/nab‐paclitaxel regimen as first‐line chemotherapy. After 10‐month treatment, PET‐CT revealed progression disease (PD). Since February 2019, he received 2 cycles of FOLFIRI. However, the patients underwent rapid PD. Then, he received FOLFIRINOX, which included oxaliplatin (a platinum‐based drug), as third‐line therapy, and follow‐up images revealed partial response (PR). Mutational profiling analysis using targeted NGS revealed that the patient carried a germline *PALB2* variant. Olaparib (a PARP inhibitor) was thereby administered as maintenance therapy. Surprisingly, he showed a significant response to olaparib, and a PFS of 23 months was observed. This case indicated that PDAC patients with germline *PALB2* variants may derive benefits from both platinum‐based chemotherapy and PARP inhibitor.

## 4 DISCUSSION

Alterations in HRR genes are emerging as novel targets for treatment in different cancers and, particularly, for personalized therapies. In the present study, we performed germline testing by next‐generation sequencing using a multigene panel of the 21 HRR genes in 256 Chinese PDAC patients. Using this expanded gene panel, we found that homologous recombination deficiency in PDAC may be underreported. In our study, when restricting our analysis to *BRCA1/2* variants, we found that only 1.6% of patients are represented. When including non‐*BRCA* HRR variants, this number increased to 7.0%. Multigene panel testing could identify HR defects in an additional 5.5% of patients, expanding the population who may benefit from DNA damage response agents. The overall incidence of germline variants in our cohort was similar to what has been reported in previous studies. Yurgelun et al. found that 7.3% of patients carried the germline variants in 24 detected DNA damage response and repair (DDR) genes in an American PDAC cohort (Yurgelun et al., 2019). Other studies have demonstrated that 3.9%–19.8% of patients with PDAC carry germline P/LPVs in cancer‐predisposing genes (Brand et al., 2018; Cremin et al., 2020; Hu et al., 2018; Shindo et al., 2017; Yurgelun et al., 2019). However, we found the differences in variant rates among studies mainly caused by gene panel involvement and ethnic specificity.

A systematic review included 60 studies with 21,842 participants who reported that the main contribution to homologous recombination deficiency (HRD) was through *BRCA2*, *BRCA1*, and *ATM*, followed by *FANC* genes, *CHEK2*, and *PALB2* in PDAC patients (Casolino et al., 2021). Based on the Chinese population, we found that the most frequently deleterious mutated genes were *ATM*, *BRCA2*, and *PALB2*. However, differences were found in the mutation spectrum among different population cohort. *BRCA1* deleterious variant was more prevalent in non‐hispanic white and Greek PDAC population (0.65% and 0.4%, respectively), but it was not found in our cohort study.

Currently, little is known about the molecular differences that might exist between PDAC patients with mutated HRR genes and those with wild‐type HRR genes. In our study, we analyzed the association between germline HRR variants and tumor *KRAS* status. However, no significant correlation was observed. Seeber A et al. presented a large study investigating the molecular landscape of patients with *BRCA*‐mutated and *PALB2*‐mutated pancreatic cancer. No differences were found in the prevalence of *KRAS* alterations. However, patients with *BRCA/PALB2* variants were associated with the expression of biomarkers that are potentially associated with response to immunotherapy such as tumor mutation burden (TMB), PD‐L1 expression, and microsatellite instability (MSI) status (Seeber et al., 2020).

Previous research showed that PDAC patients belonging to families with known *BRCA1/2* variants are a decade younger (Golan et al., 2014). There is no unified definition regarding the age of early‐onset pancreatic cancer. In our study, a cut‐off of 50 years old was used according to several previous research (Okur & Chung, 2017; Brune et al., 2010). We found that the HRR gene variant rate was similar between early‐onset and non‐early‐onset patients. Although there was a slightly higher variant rate in PDAC patients with a family history of cancer or multiple cancer history, no significant difference was observed (22.2% vs. 6.9%, P = 0.137; 15.0% vs. 6.8%, P = 0.176). A previous study also reported that the rate of germline variants in HRD genes is similar in sporadic and familial PDAC patients (Casolino et al., 2021). These findings highlighted that onset of age, family history, and history of multiple cancer were not predictors of an underlying genetic factor in PDAC patients. Therefore, current recommendations are that all newly diagnosed patients should undergo germline testing, particularly as a subset of these variants are therapeutically actionable (Lowery et al., 2018).

In our study, we identified *PALB2* as one of the most frequently mutated genes among germline HRR genes in PDAC patients. *PALB2* encodes a protein essential for double‐strand break repair and homologous recombination by serving as a bridging molecule, which connects the BRCA complex and stimulates the strand invasion of RAD51. Recently, a study investigating rucaparib, a PARP inhibitor, showed promising efficacy in PDAC patients with *PALB2* variants. In our study, we observed one patient with *PALB2* germline mutation who exhibited a long‐time response to platinum‐based chemotherapy and PARP inhibitor. The patient remained progression‐free on PARP inhibitor therapy for nearly 2 years. This case indicated that metastatic PDAC patients who harbor a germline *PALB2* variant may benefit from PARP inhibitor. While we cannot conclusively state that the entirety of patients with HRR variants would derive clinical benefit from PARP inhibition, the predictive value of non‐*BRCA* HRR variants for PARP inhibition is not well established and warrants continued exploration.

We acknowledge that there are several limitations in the present study. With a relatively small sample size and retrospective design of this study, any conclusion should be declared with caution. Further, prospective and larger studies are needed to explore the predictive and prognostic role of HRR deleterious variants. In addition, annotations of variants in HRR genes other than *BRCA1/2* are seriously inadequate. As a result, more than 20% of patients were identified as VUS carriers. This type of inconclusive result is a hard challenge to face, requiring further functional experiments and pedigree analysis for validation.

## 5 CONCLUSIONS

In the present study, we confirmed the importance of sequencing germline HRR genes in PDAC patients. Prospective studies evaluating the prognostic and predictive role of germline HRR gene variants in PDAC are warranted.

## ACKNOWLEDGEMENTS

We sincerely thank all the participants who volunteered to take part in this study.

## AUTHORS’ CONTRIBUTIONS

All the authors have accepted responsibility for the entire content of this submitted manuscript and approved submission. B.W., W.G., and H.J. made the concept. H.J. and F.H. designed the experiments. H.J., F.H., and X.C. collected clinical samples. H.J., F.H., X.C., L.Z., and M.S. performed experiments, analyzed data, and interpreted the results. H.J. and F.H. drafted the manuscript. B.P., B.W., and W.G. edited the manuscript.

## FUNDING INFORMATION

This study was supported by the Science and Technology Commission of Shanghai Municipality (21YF1440200), the National Natural Science Foundation of China (81972000, 82172348, 81902139), Zhongshan Hospital (2018ZSLC05, 2020ZSLC54, 2021ZSQN37), the Constructing Project of Clinical Key Disciplines in Shanghai (shslczdzk03302), and the Key Medical and Health Projects of Xiamen (YDZX20193502000002).

## CONFLICT OF INTEREST STATEMENT

The authors have no conflicts of interest to declare.

## Data Availability

The data that support the findings of this study are available on request from the corresponding author.
